# Puerarin inhibited oxidative stress and alleviated cerebral ischemia-reperfusion injury through PI3K/Akt/Nrf2 signaling pathway

**DOI:** 10.3389/fphar.2023.1134380

**Published:** 2023-05-22

**Authors:** Qianqian Zhang, Min Yao, Jiajia Qi, Rui Song, Lei Wang, Jiacheng Li, Xian Zhou, Dennis Chang, Qi Huang, Lili Li, Ning Wang

**Affiliations:** ^1^ Department of Pharmacy, Anhui University of Chinese Medicine, Hefei, China; ^2^ Anhui Province Key Laboratory of Research & Development of Chinese Medicine, Anhui University of Chinese Medicine, Hefei, China; ^3^ Anhui Province Key Laboratory of Chinese Medicinal Formula, Anhui University of Chinese Medicine, Hefei, China; ^4^ Institute for Pharmacodynamics and Safety Evaluation of Chinese Medicine, Anhui Academy of Traditional Chinese Medicine, Hefei, China; ^5^ Anhui Province Key Laboratory of Pharmaceutical Preparation Technology and Application, Anhui University of Chinese Medicine, Hefei, China; ^6^ National Institute of Complementary Medicine, Western Sydney University, Westmead, NSW, Australia

**Keywords:** cerebral ischemia reperfusion injury (CIRI), puerarin, hippocampal neurons, oxidative stress, PI3K/Akt/Nrf2 pathway

## Abstract

**Introduction:** Puerarin (PUE) is a natural compound isolated from Puerariae Lobatae Radix, which has a neuroprotective effect on IS. We explored the therapeutic effect and underlying mechanism of PUE on cerebral I/R injury by inhibiting oxidative stress related to the PI3K/Akt/Nrf2 pathway *in vitro* and *in vivo*.

**Methods:** The middle cerebral artery occlusion and reperfusion (MCAO/R) rats and oxygen-glucose deprivation and reperfusion (OGD/R) were selected as the models, respectively. The therapeutic effect of PUE was observed using triphenyl tetrazolium and hematoxylin-eosin staining. Tunel-NeuN staining and Nissl staining to quantify hippocampal apoptosis. The reactive oxygen species (ROS) level was detected by flow cytometry and immunofluorescence. Biochemical method to detect oxidative stress levels. The protein expression related to PI3K/Akt/Nrf2 pathway was detected by using Western blotting. Finally, co-immunoprecipitation was used to study the molecular interaction between Keap1 and Nrf2.

**Results:** *In vivo* and vitro studies showed that PUE improved neurological deficits in rats, as well as decreased oxidative stress. Immunofluorescence and flow cytometry indicated that the release of ROS can be inhibited by PUE. In addition, the Western blotting results showed that PUE promoted the phosphorylation of PI3K and Akt, and enabled Nrf2 to enter the nucleus, which further activated the expression of downstream antioxidant enzymes such as HO-1. The combination of PUE with PI3K inhibitor LY294002 reversed these results. Finally, co-immunoprecipitation results showed that PUE promoted Nrf2-Keap1 complex dissociation.

**Discussion:** Taken together, PUE can activate Nrf2 via PI3K/Akt and promote downstream antioxidant enzyme expression, which could further ameliorate oxidative stress, against I/R-induced Neuron injury.

## 1 Introduction

Stroke is one of the most common cerebrovascular diseases and is usually divided into hemorrhagic stroke and ischemic stroke (IS), among which IS is the main type of stroke accounting for more than 85% of the stroke incidence, which also brings a heavy burden to society (Vaidya et al., 2018; Li et al., 2020). Therefore, finding novel therapeutic medicines for IS is of great significance for human health.

Insufficiency of blood supply or embolic events may cause IS, whereas reperfusion pressure to restore blood supply to the ischemic area can further cause cerebral ischemia-reperfusion (I/R) injury (Albrecht et al., 2019; Luo et al., 2019). The pathogenesis of I/R is complex and diverse, including oxidative stress, energy metabolism disorder, disruption of ion homeostasis, calcium overload, neuron overexcitability, inflammation, *etc,.* (Benjamin et al., 2017; Stanaway et al., 2018). Among them, massive reactive oxygen species (ROS) are generated caused of the secondary supply of glucose and oxygen, the overproduced ROS destroys the endogenous redox balance, and thus oxidative stress occurs and ultimately causes neuronal death or apoptosis (Sun et al., 2018; Su et al., 2020). Therefore, upregulating the expression of antioxidant proteins or inhibiting the production of free radicals becomes to be the main strategy to treat I/R.

Nucleus factor-erythroid 2-related factor 2 (Nrf2) is a key transcription factor, which can maintain cell homeostasis in the human antioxidant system, and plays an indispensable role in the normal operation of the body (Pang et al., 2016; Zhang et al., 2017). Numerous studies have confirmed that PI3K/Akt is responsible for cell survival, migration and proliferation (Ramos et al., 2017), while promoting Nrf2 free into the nucleus and regulating the level of downstream antioxidant enzymes (Lu et al., 2014; Chen et al., 2015; Tongqiang et al., 2016; Li et al., 2018), thus shows a neuroprotective role in ischemia/reperfusion (I/R).

*Puerarialobata (Willd.) Ohwi* is one of the most common traditional Chinese medicine in clinical use, and puerarin (PUE) is its main bioactive component (Wong et al., 2011). Due to its low toxicity and high safety, PUE has a unique effect on the treatment of cardio-cerebrovascular diseases (Wei et al., 2014; Gao et al., 2019). Studies have shown that the neuroprotective mechanism of PUE on ischemic brain injury may be related to increasing the activity of antioxidant enzymes and reducing lipid peroxidation (Chang et al., 2009; Tao et al., 2017). However, the mechanism by which PUE attenuates I/R injury by inhibiting oxidative stress is not clear.

So, we first validated the protective effect of PUE on I/R. And these effects might be mediated by reducing oxidative stress and apoptosis via PI3K/Akt to promote the entry of Nrf2 into the nucleus. This study provides a new idea for PUE to mitigate I/R injury and exert a cerebral protective effect, which supplied a research basis for exploring potential new active ingredients of TCM in the treatment of stroke.

## 2 Materials and methods

### 2.1 Chemicals and reagents

PUE (No: MUST-21010610, purity ≥98%) from Mansite Co., Ltd. (Chengdu, China), Dulbecco’s Modified Eagle’s Medium (DMEM)was purchased from Hyclone (Shanghai, China); 10% fetal bovine serum (FBS, Kibbutz Beit Haemek, Israel) and Triphenyl tetrazolium chloride (TTC) was purchased from Sigma Chemical Co (No: B2731St). Malondialdehyde (MDA, No: A003-1-1), Superoxide dismutase (SOD, No: A001-3-1), Glutathione peroxidase (GSH-px, No: A005-1-2), Glutathione (GSH, No: A006-2-1), Catalase (CAT, No: A002-1-1) (Nanjing Jian Cheng Bioengineering Institute); lactate dehydrogenase (LDH, No: C1007) and reactive oxygen species (ROS, No: S0033) assay kits were all purchased from Bestbio Co., Ltd. (Shanghai, China). The antibodies against PI3K, p-PI3K, Akt, p-Akt, Nrf2, Keap1, and HO-1 were all provided by Abcam (Cambridge, MA, United States). The antibody against GAPDH was purchased from CST.

### 2.2 The protective effect of PUE on I/R *in vivo*

#### 2.2.1 Animals and establishment of MCAO/R model

Male SD rats (weight: 220–240 g) were provided by Hunan Silaike Experimental breeding Co., Ltd. (Certificate No. SCXK (Xiang) 2019-0004). These rats could get food and water at will and were fed adaptively for 3 days before the experiment. After anesthesia, the *in vivo* (MCAO/R) model of experimental rats was established according to the previous studies (Wu et al., 2020). Tweezers were used to separate the left carotid artery and internal carotid artery, then a waxed thread was inserted to block the middle cerebral artery (MCA), and 2 h later it was pulled out to the site of MCAO/R injury for reperfusion. All the above animal experiments have been approved by the Experimental Animal Ethics Committee of Anhui University of Traditional Chinese Medicine (Hefei, Anhui, China; animal ethics code: AHUCM-RATS-2022027; 19 September 2022).

#### 2.2.2 Grouping and administration

To study the therapeutic effect of PUE on MCAO/R model rats, the experimental rats with successful model establishment were randomly grouped: model group, edaravone group (a positive drug), PUE low-dose group (PUE-L, 25 mg/kg/day, dissolved in 10% methyl glycol, diluted with saline), PUE medium-dose group (PUE-M, 50 mg/kg/day), PUE high-dose group (PUE-H, 100 mg/kg/day) and ten rats for each group. The administration of PUE used in this study was performed based on a previous study (Xu et al., 2005; Tao et al., 2017). The sham operation group did the same operation, but did not establish the MCAO/R model. Rats in PUE (Xie et al., 2014; Wang et al., 2018) and edaravone (30 mg/kg/day) (Luan et al., 2020) groups received drug once a day for 7 days.

The mechanism of action of PUE is grouped as follows: model group, PUE (100 mg/kg), LY294002 (a PI3K inhibitor), PUE (100 mg/kg) + LY294002 groups, another ten rats were selected as sham operation group did the same operation but not occluded. LY294002 (10 mg/kg/day, dissolved in DMSO) (Tao et al., 2017) was administered by intraperitoneal injection 30 min before ischemia. The other groups were given PUE once a day for 7 days.

#### 2.2.3 Longa assay and TTC staining

The neurobehavior after 72 h of MCAO/R was scored with reference to Longa’s modified scoring method (Longa et al., 1989). The scoring criteria were: 0, normal behavior, no obvious injury; 1, forepaw could not be fully extended and was flexed; 2, circling around one side of the tail chase; 3, difficulty walking and hemiparesis on one side; 4, no spontaneous walking.

Fresh brain tissue was removed and frozen at −20°C for 20–30 min, 2 mm thick coronal sections were made, stained with 2,3,5-triphenyltriazole sodium chloride (TTC), incubated at 37°C for 20 min, and the whole process needed to be operated away from light. 4% paraformaldehyde was soaked overnight and photographed to calculate brain infarct volume: infarct area × average section thickness (2 mm), and analyzed by ImageJ.

#### 2.2.4 Hematoxylin and eosin (HE) staining, nissl staining, and TUNEL-NeuN assay

Fresh brain tissue from the ischemic area of each group of rats was taken, paraffin-embedded 4-μm-thick sections were made, stained with HE dye, and images of cell morphology were observed under the microscope. Prepared tissue sections were soaked in 0.1% cresyl violet at 37°C for 20 min, rinsed and dehydrated and placed under a microscope for observation. This experiment mainly assessed the morphological changes of neurons in the CA1 region of the hippocampus, with round and pale staining-like nuclei of normal cells. The final data were expressed as the number of surviving cells/field.

The preparation of tissue section is the same as above. After staining with Nissl dye, the morphological changes of Nissl bodies were observed under light microscope. The number of normal and stained cells was calculated by ImageJ software to evaluate neuronal damage.

The brain tissues of each group were fixed, dehydrated and embedded in sections, dewaxed, repaired with broken membranes, and the samples were treated with reagent 1 (TdT) and reagent 2 (dUTP) respectively and then blocked with endogenous peroxidase, then stained with reagent 3 (converter-POD), stained with diaminobenzidine, and finally the nuclei were re-stained with hematoxylin, dehydrated and sealed and ready to be placed under the microscope for observation.

#### 2.2.5 Determination of brain tissue SOD, MDA, GSH, GSH-px, and CAT

The hippocampal tissues of different grouped ischemic areas were homogenized in cold saline at pH 7.0 to prepare 10% w/v brain tissue homogenates. The oxidative stress levels were detected by referring to the instructions of SOD, GSH-px, GSH, MDA and CAT kits, respectively (Nanjing Jiancheng Institute of Biological Engineering).

#### 2.2.6 Immunohistochemistry

Each group of brain tissue was dewaxed and sectioned before the antigen was extracted. Paraffin sections were incubated in 5% goat serum for 15–20 min and then incubated in Nrf2 antibody for 24 h, 4°C. The next day the sections were incubated with HRP polymer at RT for 30 min protected from light, and finally stained with DAB mixture for 5–10 min and observed under microscope.

### 2.3 Neuroprotective effect of PUE *in vitro*

#### 2.3.1 Cell culture and OGD/R model

Mouse hippocampal-derived HT22 cells were cultured in an incubator. The medium consisted of 100 U/mL penicillin, streptomycin, and 10% fetal bovine serum at 5% CO_2_, 37°C humidity. The growth medium was replaced every day. OGD/R was used to simulate I/R. The complete medium in HT22 cells was replaced with glucose-free DMEM and placed in a 5% CO_2_/95% N_2_ incubator for 2 h. At the end of the incubation period, the cells were switched back to normal DMEM and restored to oxygen and glucose for 24 h (Li et al., 2020).

#### 2.3.2 Experimental grouping *in vitro*

The *in vitro* experiments were grouped as follows: Blank control, OGD/R (Model) group, OGD/R + PUE (150 nM), OGD/R + PUE (200 nM), OGD/R + PUE (250 nM) and OGD/R + edaravone (10 μM) groups; To explore whether the protective effects PUE on HT22 cells were related to PI3K/Akt/Nrf2 pathway, the grouping was as follows: Blank control, OGD/R (Model) group, OGD/R + PUE (250 nM), OGD/R + LY294002 (20 μM), OGD/R + LY294002+ PUE (250 nM) groups. hippocampal neurons were pretreated with LY294002 (Wu et al., 2020), 3 h before OGD/R.

#### 2.3.3 Cell viability and LDH assay

According to the instructions of CCK-8 kit, HT22 cells in 96-well plates were treated with mold-making administration, then diluted CCK-8 reagent was added and incubated in an incubator protected from light for 1 h. The cells were detected on the machine at 450 nm with an enzyme marker. According to the OD value of each group, HT22 cell viability was calculated by substituting into the formula.

When the cells were stimulated, the intracellular LDH enzyme would permeate into the culture medium through the damaged cell membrane, so the LDH activity assay kit was selected to detect the cytotoxicity. The supernatant of the cells was collected and centrifuged after modeling and administration. According to the instructions of the kit, the activity was determined by spectrophotometry under the condition of wavelength 340 nm.

#### 2.3.4 Hoechst 33258 and Annexin V-FITC-PI

The apoptosis of HT22 cells was detected by Hoechst 33258 fluorescence staining and flow cytometry. In the Hoechst 33258 experiment, HT22 was inoculated in a 6 well plate for 24 h, and then stained for 15 min with 2 μg/mL Hoechst 33258 (2 μg/mL) according to the instructions. Observation of cells by fluorescence microscope.

HT22 (1 × 10^6^ cells/well) were collected. HT22 was re-suspended in 500 mL binding buffer and rinsed for 3 times. The cells were evenly stained with 5 μL FITC-Annexin V and incubated with 10 μL propidium iodide (PI) for 20 min before going on the machine. FlowJo software is used for data processing (FlowJo, version 10.0).

#### 2.3.5 ROS asssy

HT22 cells were treated with a series of concentrations of PUE after OGD/R. After the treatment, 30 min was fixed with 4% paraformaldehyde, 30min was incubated with 10 μg/mL ROS working solution (China Biyuntian Biotechnology Co., Ltd.) at 37°C, and finally PBS was washed three times. Then observed under fluorescence microscope.

The level of ROS in HT22 cells was detected by flow cytometry. HT22 cells (3 × 10^5^ cells/well) were inoculated in 6 well plates. After the treatment of model administration, the cell precipitation was re-suspended in the working solution diluted in 10 μM DCFH-DA solution, and 20 min was incubated without light. Finally, flow cytometry was used to detect it. FlowJo software is used for data processing.

#### 2.3.6 Oxidative stress measurements by biochemical method

HT22 cells were homogenized by RIPA buffer, and proteins were collected. The levels of MDA, SOD, CAT, GSH and GSH-px in HT22 cells were detected according to the instructions of biochemical kits provided by biotechnology companies.

#### 2.3.7 Western blot

Cells or tissues were lysed and proteins were separated from them, and then the concentrations were determined according to the BCA kit (Bestbio, China). After adding 20–30 μg of the above protein, the membrane was separated by electrophoresis using SDS-PAGE for 2 h, and then transferred to polyvinylidene fluoride (PVDF) membrane for about 90 min. The membrane was then immersed in 5% skimmed milk for about 2 h and operated at room temperature. Finally, incubated with primary antibodies including PI3K (1:1,000), p-PI3K (1:1,000), Akt (1:1,000), p-Akt (1:1,000), Nrf2 Nucleus (1:2,000), Nrf2 Cytoplasm (1:2000), HO-1 (1:1,000), Keap1 (1:1,000), GAPDH (1:5,000) and Lamin B1 (1:2,000) at 4°C, respectively. The next day transfer to Rabbit antibody solution (1:10,000) and incubate for 2 h. GAPDH acts as an internal protein reference. Membranes were incubated in enhanced chemiluminescence (ECL) (Thermo, United States) solution. AmershamImager 600 (GE,United States) can detect the expression of individual proteins on membranes.

#### 2.3.8 The interaction between Keap1 and Nrf2 was verified by immunoprecipitation

HT22 cells were homogenized, lysed, and centrifuged to extract total protein. The BCA method was used for protein quantification. Antibody/IgG-Beads complex and target protein-Antiton-Beads complex were prepared to detect the binding and dissociation of keap1 and nrf2 were detected by Western blotting to verify the molecular interaction.

### 2.4 Statistical analysis

The experimental data were expressed as means ± standard deviation (SD). The results were analyzed mainly by one-way ANOVA and *t*-test. When *p* < 0.05, the results proved to be statistically significant.

## 3 Results

### 3.1 PUE reduced neurological scores and alleviated neuronal damage

**FIGURE 1:**
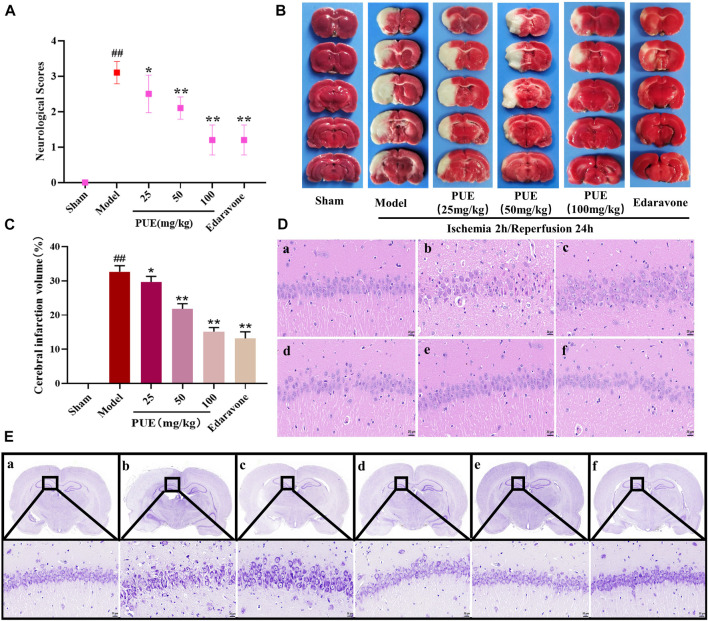
PUE reduced neurological scores and alleviated neuronal damage in MCAO/R rat brains. **(A)** PUE reduced the neurological score assessed by the Zea Longa test after cerebral I/R (*n* = 10). **(B)** Representative images of TTC staining. **(C)** Quantitative analysis of cerebral infarction volume (*n* = 3). **(D)** The morphological alterations of cells in the hippocampal CA1 region were measured by HE staining (×400). **(E)** The morphological alterations of cells in the hippocampal CA1 region were examined by Nissl staining (×400). **(a)** Sham group; **(b)** model group; **(c)** PUE (25 mg/kg) group; **(d)** PUE (50 mg/kg) group; **(e)** PUE (100 mg/kg) group; **(f)** edaravone. The scale bar was 20 µm. All data were expressed as mean ± SD. ^##^ *p* < 0.01 vs. sham group, *p < 0.05 or **p < 0.01 vs. model group.

The results of neurological score, cerebral infarction volume, and morphological changes showed that PUE (100 mg/kg) could improve the neurological score (Figure 1A) and infarct volume (Figures 1B, C). The model group of hippocampal neuronal cells had a disordered arrangement of nucleoli disappearance and partial nucleoplasm atrophy. However, this tendency could be reversed by PUE (Figure 1D). In the model group, the Nissl bodies in the hippocampal CA1 region were obviously abnormal, most of the nucleoli disappeared and the intercellular space became larger. Nevertheless, PUE (100 mg/kg) can make the cells arrange neatly, improve the morphology of nucleolus and reduce vacuoles (Figure 1E). The above results confirm that PUE reverses the injury by MCAO/R and plays a protective role.

### 3.2 PUE reduced neuronal apoptosis in cerebral ischemia tissue

**FIGURE 2:**
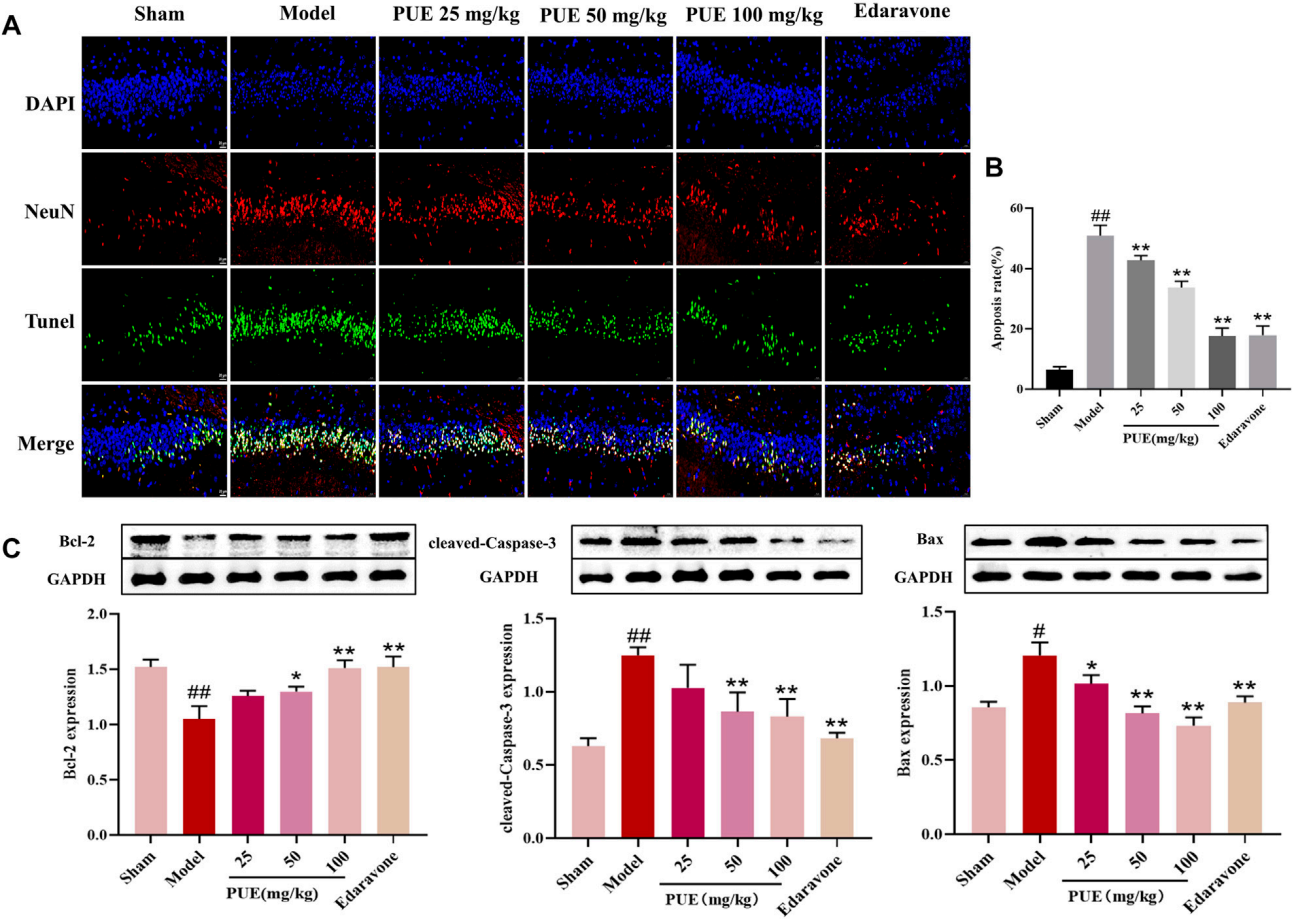
PUE alleviates the neuro apoptosis of cerebral ischemic tissue. **(A)** Tunel-NeuN staining: green, Tunel-positive staining; blue, DAPI core dye; red, neuron-specific nucleus protein (NeuN) (400×). **(B)** Quantitative analysis of the proportion of Tunel-positive neurons in each group. **(C)** Bax, Caspase-3 and Bcl-2 protein expression in rat hippocampal neurons; Quantification results of protein expression. All data are expressed as mean ± SD. ^##^ *p* < 0.01 vs. sham group, *p < 0.05 or **p < 0.01 *vs*. model group.

In order to clarify the effect of PUE on apoptosis, we performed Tunel-NeuN immunofluorescence double staining. PUE (100 mg/kg) significantly reduced the apoptosis rate, from 48.5% to 19.7% (Figures 2A, B). Then, Bax, cleaved-caspase3, and Bcl-2 were detected by Western blotting method. Briefly, in the model state, Bax and cleaved-caspase 3 were significantly increased and Bcl-2 was decreased. However the above trend was reversed by PUE (100 mg/kg) (Figure 2C). Thus, it could be inferred that PUE exerted neuron protective effects by inhibiting apoptosis.

### 3.3 PUE decreased the level of ROS and activity of enzymes related to oxidative stress levels in MCAO/R model rats’ brain tissue

**FIGURE 3:**
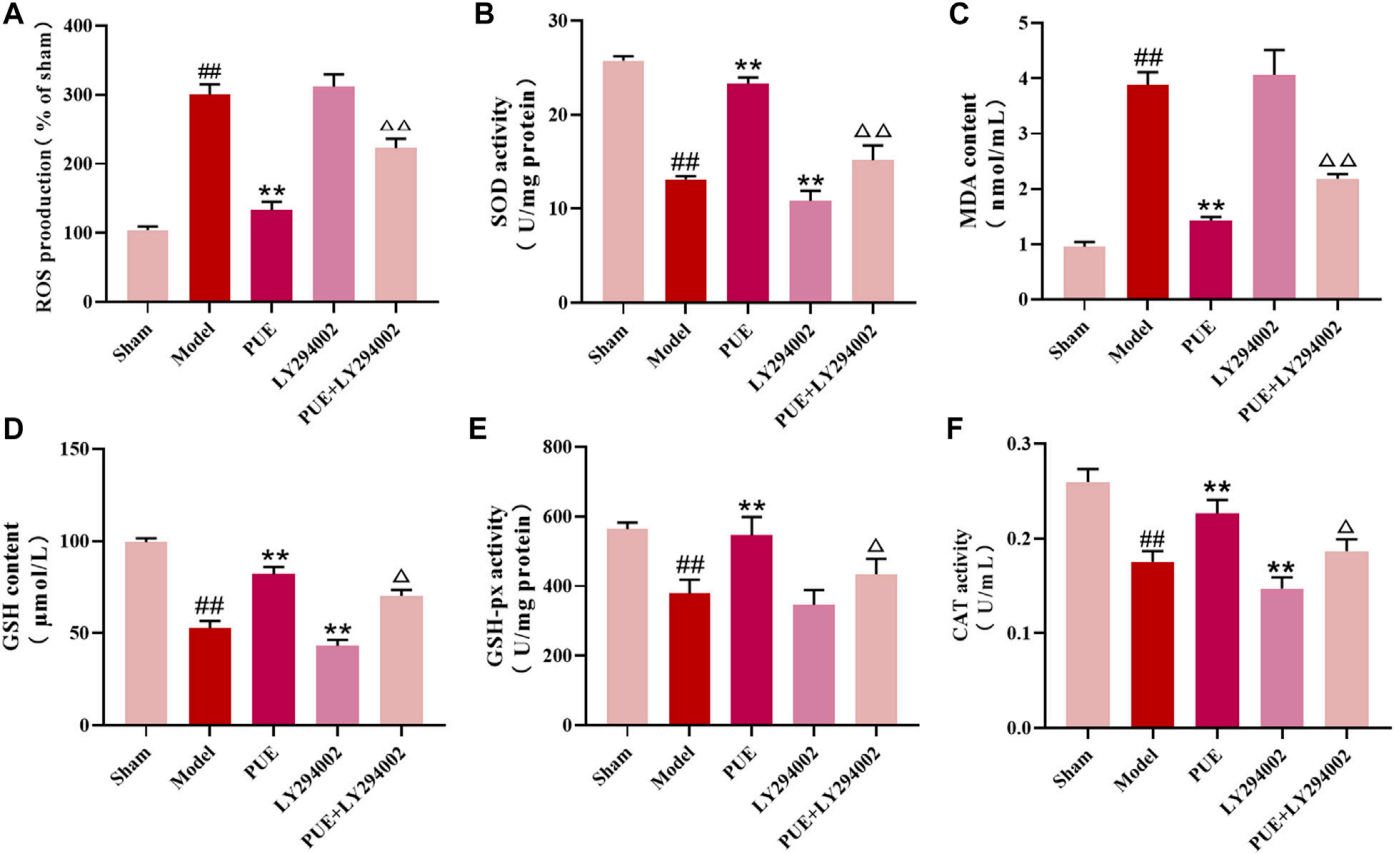
PUE regulated the oxidative stress level in the MCAO/R model. **(A–F)** The levels of ROS, SOD, MDA, GSH, GSH-px, and CAT in the brain tissues were measured by the corresponding kits. All data are expressed as mean ± SD (*n* = 6). ^##^ *p* < 0.01 vs. sham group, *p < 0.05 or **p < 0.01 vs. model group, ^△^ *p* < 0.05 or ^△△^ *p* < 0.01 vs. PUE group.

Next, we examined the level of oxidative stress in brain tissue. In the model state, the contents of antioxidant enzymes SOD, GSH, GSH-px, and CAT were reduced (*p* < 0.01), and the contents of ROS and MDA were significantly increased (*p* < 0.01). However, PUE (100 mg/kg) significantly (*p* < 0.01) reversed these changes. LY294002 inhibited the therapeutic effect of PUE. In conclusion, MCAO/R induces oxidative stress, suggesting that PUE may exert a protective effect by inhibiting oxidative stress (Figures 3A–F).

### 3.4 PUE attenuated hippocampal neuron injury after MCAO/R via PI3K/Akt/Nrf2

**FIGURE 4:**
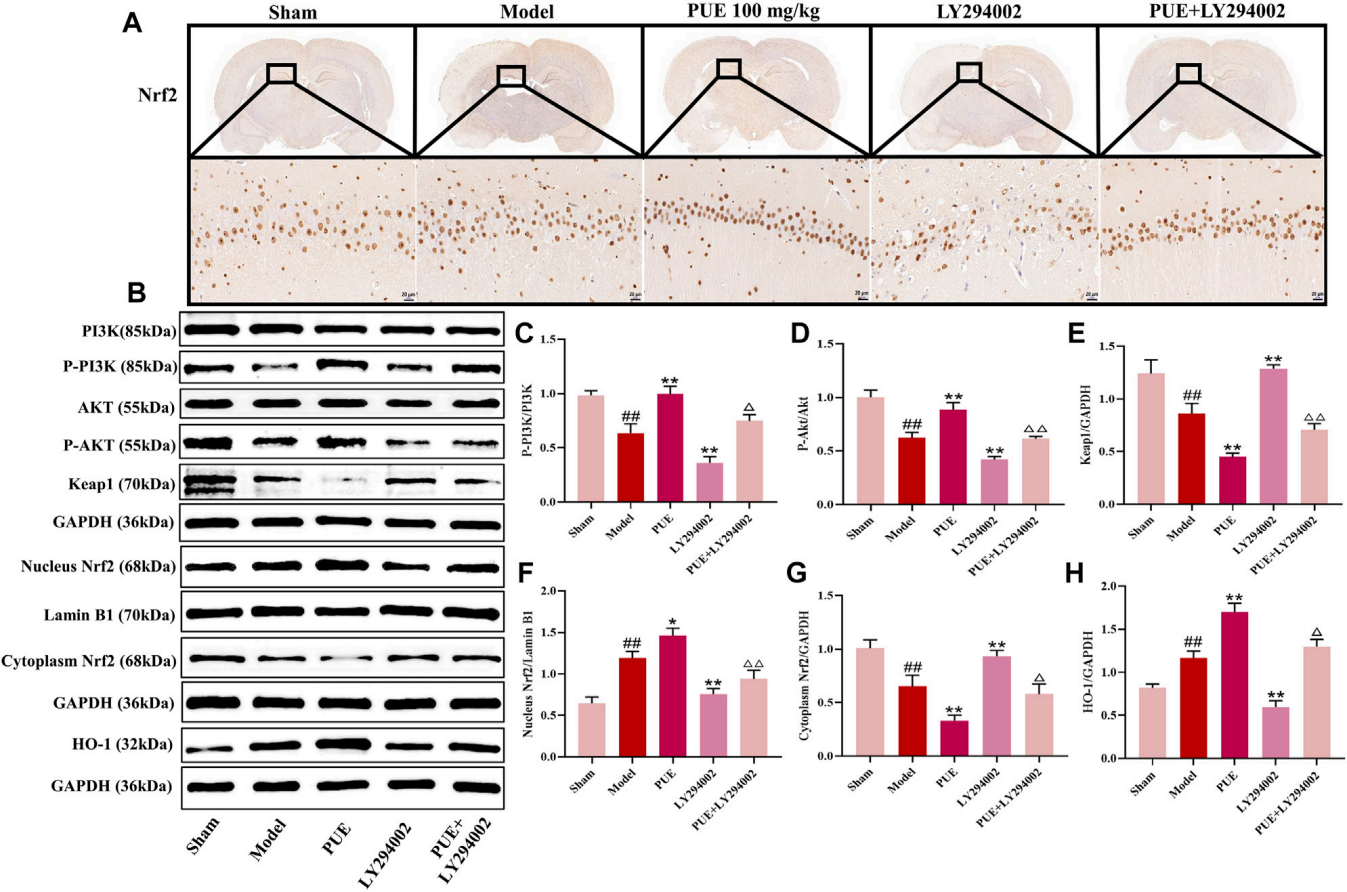
PUE attenuates hippocampal neuron injury after MCAO/R via PI3K/Akt/Nrf2. **(A)** The expression and location of Nrf2 were determined by immunohistochemistry assay (×400). **(B)** Representative protein imprinting image of PI3K, Akt, P-PI3K, P-Akt, Keap1, Nucleus Nrf2, Cytoplasm Nrf2 and HO-1 in hippocampal neurons of the ischemic side of rat brain tissue. **(C)** Quantitative analysis of p-PI3K/PI3K protein expression. **(D)** Quantitative analysis of p-Akt/Akt protein expression. **(E)** Quantitative analysis of Keap1 protein expression. **(F)** Quantitative analysis of Nucleus Nrf2 protein expression. **(G)** Quantitative analysis of Cytoplasm Nrf2 protein expression. **(H)** Quantitative analysis of HO-1 protein expression. All data are expressed as mean ± SD. ^##^ *p* < 0.01 *vs*. sham group, *p < 0.05 or **p < 0.01 vs. model group, ^△^ *p* < 0.05 or ^△△^ *p* < 0.01 vs. PUE group.

Immunohistochemical methods were used to study the state of Nrf2 in the hippocampal region of MCAO/R rats. The results showed that Nrf2 was activated in hippocampal neuronal cells and further into the nucleus (Figure 4A). As shown in Figures 4B–H, there was little change of PI3K and Akt among the pathway-related proteins, while the p- PI3k, p- Akt, cytoplasm Nrf2 and Keap1 was decreased (*p* < 0.01) and the nucleus Nrf2 and HO-1 was increased (*p* < 0.01) in the model group rats. In response to LY294002 inhibition, cytoplasm Nrf2 and Keap1 expression increased and p- PI3k, p- Akt, nucleus Nrf2, and HO-1 decreased (*p* < 0.01, *p* < 0.05). The above was reversed in the PUE treatment group.

### 3.5 PUE reduces OGD/R damage to HT22 cells

**FIGURE 5:**
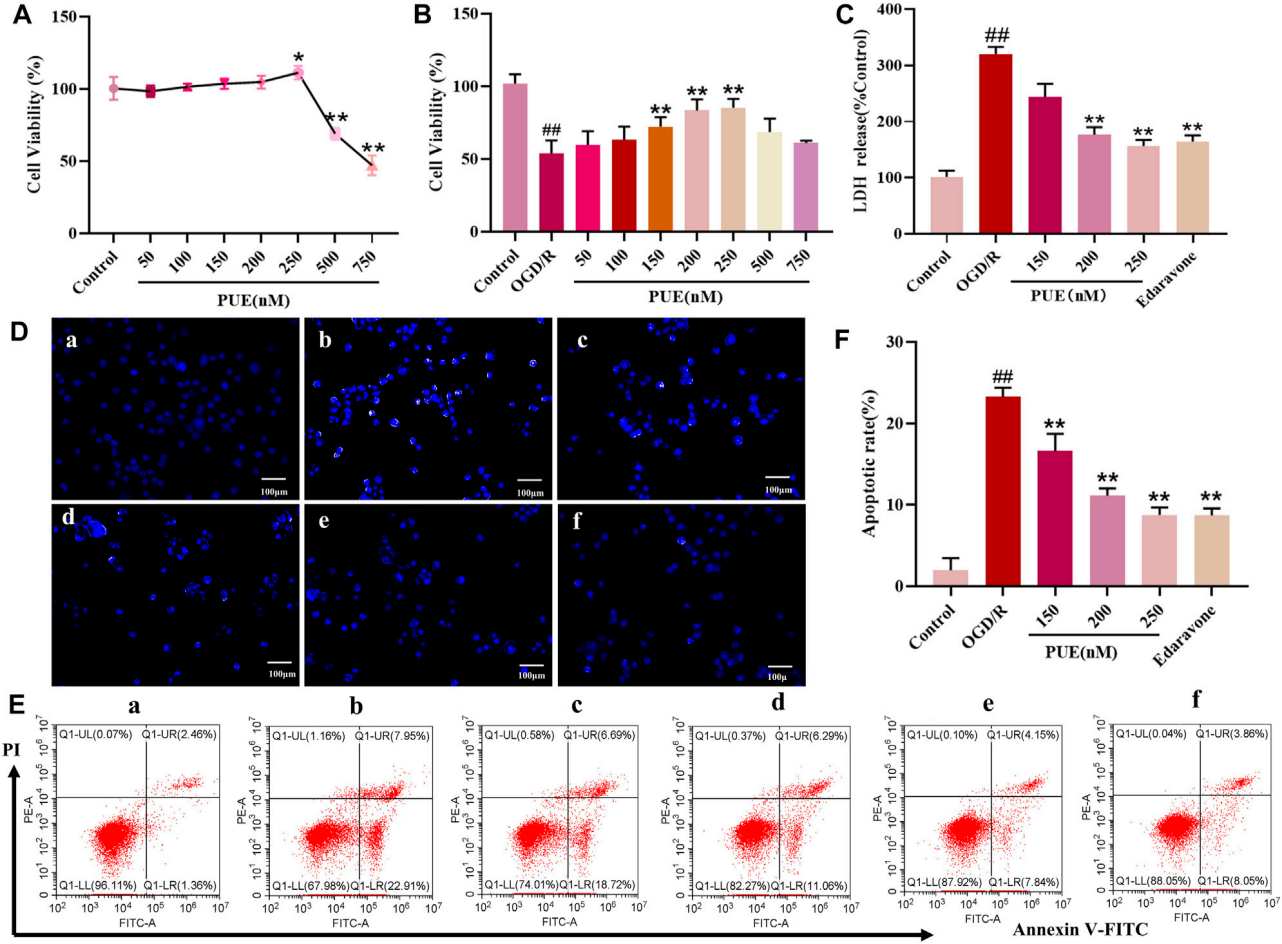
PUE promotes the viability of hippocampal neurons induced by oxygen-glucose deprivation/reperfusion (OGD/R). **(A)** Hippocampal neurons were treated with PUE at various concentrations (50–750 nM) and the cell viability was measured by the CCK-8 assay. **(B)** Cell viability was measured by the CCK-8 assay after OGD/R and normal conditions. **(C)** Effect of PUE on the release of LDH from hippocampal neurons damaged by OGD/R. **(D)** The percentage of HT22 cell apoptosis was analyzed by staining cells with Hoechst 33258 (blue represents viable cells, × 200). **(E,F)** Apoptotic cells rate were detected by flow cytometry. **(a)** Control group; **(b)** OGD/R group; **(c)** PUE (150 nM) + OGD/R; **(d)** PUE (200 nM)+OGD/R; **(e)** PUE (250 nM)+OGD/R; **(f)** edaravone. All data are expressed as means ± SD (*n* = 6). ^##^ *p* < 0.01 vs. control group, *p < 0.05 or **p < 0.01 vs. OGD/R group.

To further investigate the neuroprotective effects mechanism of PUE, the results of CCK-8 experiments showed that the drug concentration below 250 nM was in the safe and non-toxic range (Figure 5A). Also in this range PUE (250 nM) had the most significant protective effect on OGD/RHT22 cells (Figure 5B). Edaravone is a free radical scavenger, so we use edaravone (10 µM) as the positive control, and the results showed that both were comparable in efficacy. Also, PUE and edaravone administration reversed the significant increase in LDH concentration caused by OGD/R injury (Figure 5C).

Hoechst 33258 could penetrate the damaged cell nuclei and label the nuclei in the early stage of apoptosis. Obviously, most of the control group HT22 cells emitted weak blue fluorescence, which turned into strong blue fluorescence after OGD/R, and the intensity of blue fluorescence was significantly reduced after PUE treatment (Figure 5D). Subsequently, the apoptosis rate was quantified by Annexin V/PI staining, and the apoptosis rate increased to 19.73% when HT22 cells were in the OGD/R state, while PUE (150, 200 and 250 nM) treatment decreased the apoptosis rate to 15.58%, 13.53% and 11.21% (*p* < 0.01, *p* < 0.05). Taken together, PUE attenuated HT22 cell damage by inhibiting the apoptosis rate (Figures 5E, F).

### 3.6 PUE inhibits ROS overproduction of HT22 cells induced by OGD/R

**FIGURE 6:**
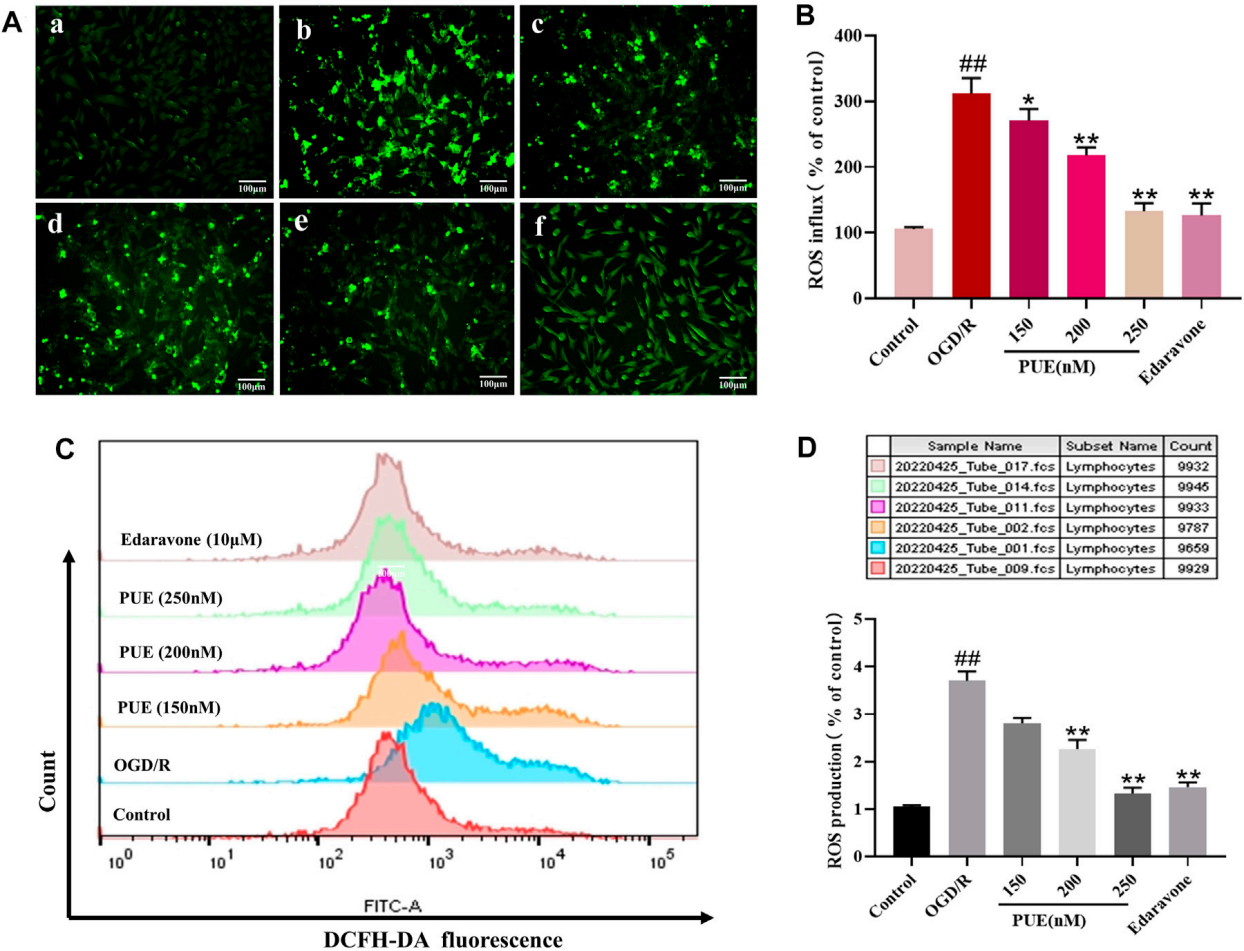
The effect of PUE on oxygen-glucose deprivation/reperfusion (OGD/R)-induced ROS release of hippocampal neurons. **(A)** The reactive oxygen species (ROS) level induced by OGD/R was detected by fluorescence microscopy (×200). The mean of fluorescence intensity is shown in **(B)**. **(C)** ROS generation was determined with DCFH-DA staining and analyzed by Flow Jo. The mean of fluorescence intensity is shown in **(D)**. **(a)** Control group; **(b)** OGD/R group; **(c)** PUE (150 nM) + OGD/R; **(d)** PUE (200 nM) + OGD/R; **(e)** PUE (250 nM) + OGD/R; **(f)** edaravone. All data are expressed as means ± SD (*n* = 3). *p < 0.05 or **p < 0.01 vs. OGD/R group.

The percentage of DCFH-DA fluorescence intensity was used as a criterion to determine the rate of ROS production, so the DCFH-DA fluorescent probe was used to detect the expression of ROS in HT22 cells. The fluorescence results showed that PUE (250 nM) administration significantly reduced the production of reactive oxygen species (*p* < 0.01), and edaravone was comparable to its effect (Figures 6A, B). Flow cytometry results were consistent with these results, confirming that PUE (250 nM) was able to reduce the level of OGD/R-induced ROS generation (Figures 6C, D).

### 3.7 PUE changed the level of enzymatic activity related to oxidative stress

**FIGURE 7:**
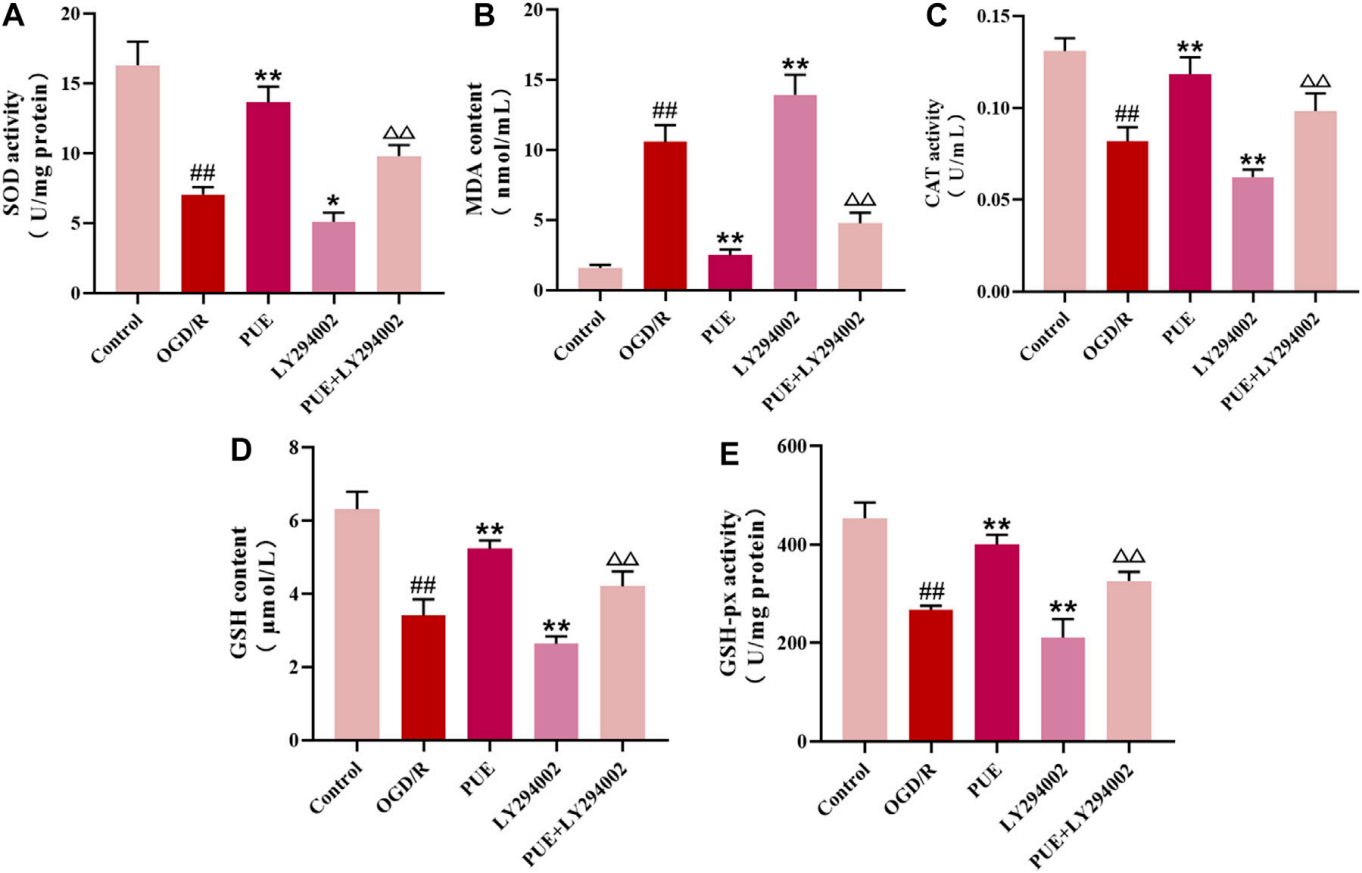
PUE changed the level of enzymatic activity related to oxidative stress in HT22 cells. **(A-E)** The levels of SOD, MDA, CAT, GSH, and GSH-px were measured by the corresponding kits. All data are expressed as mean ± SD (*n* = 6). ^##^ *p* < 0.01 vs. control group, *p < 0.05 or **p < 0.01 *vs.* OGD/R group, ^△^p < 0.05 or ^△△^ *p* < 0.01 *vs.* PUE group.

As seen in Figure 7, PUE (250 nM) significantly reversed the decrease in SOD, CAT, GSH-px, and GSH levels along with the increase in MDA levels after OGD/R (*p* < 0.01, *p* < 0.05), while the therapeutic effect of PUE was limited by the use of LY294002. In conclusion, OGD/R induced oxidative stress, suggesting that PUE may exert a protective effect by inhibiting oxidative stress.

### 3.8 PUE attenuates hippocampal neuron injury after OGD/R via PI3K/Akt/Nrf2

**FIGURE 8:**
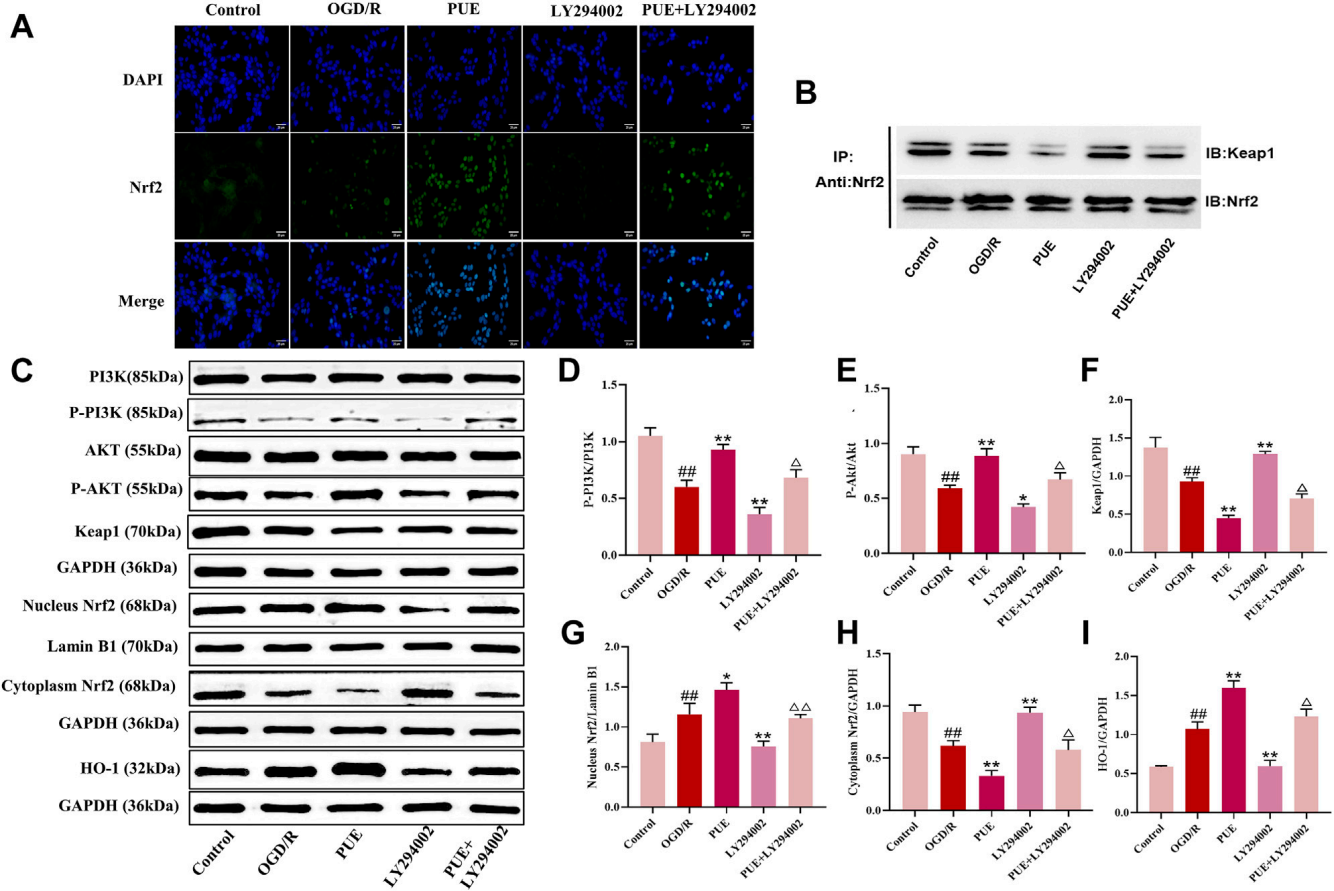
PUE affects the expression of proteins related to PI3K/AKT/Nrf2. **(A)** The expression of NRF2 (green labeled) and nuclear (blue labeled) in HT22 cells was measured by confocal laser scanning microscope. Bar = 25 μm. **(B)** Co-immunoprecipitation evaluation of Nrf2-Keap1 interaction in HT22 cells. **(C)** Representative protein imprinting image of PI3K, Akt, P-PI3K, P-Akt, Keap1, Nucleus Nrf2, Cytoplasm Nrf2 and HO-1 in hippocampal neurons of the ischemic side of rat brain tissue. **(D)** Quantitative analysis of p-PI3K/PI3K protein expression. **(E)** Quantitative analysis of p-Akt/Akt protein expression. **(F)** Quantitative analysis of Keap1 protein expression. **(G)** Quantitative analysis of Nucleus Nrf2 protein expression. **(H)** Quantitative analysis of Cytoplasm Nrf2 protein expression. **(I)** Quantitative analysis of HO-1 protein expression. All data are expressed as mean ± SD. ^##^ *p* < 0.01 vs. control group, *p < 0.05 or **p < 0.01 vs. OGD/R group, ^△^ *p* < 0.05 or ^△△^ *p* < 0.01 vs. PUE group.

**FIGURE 9:**
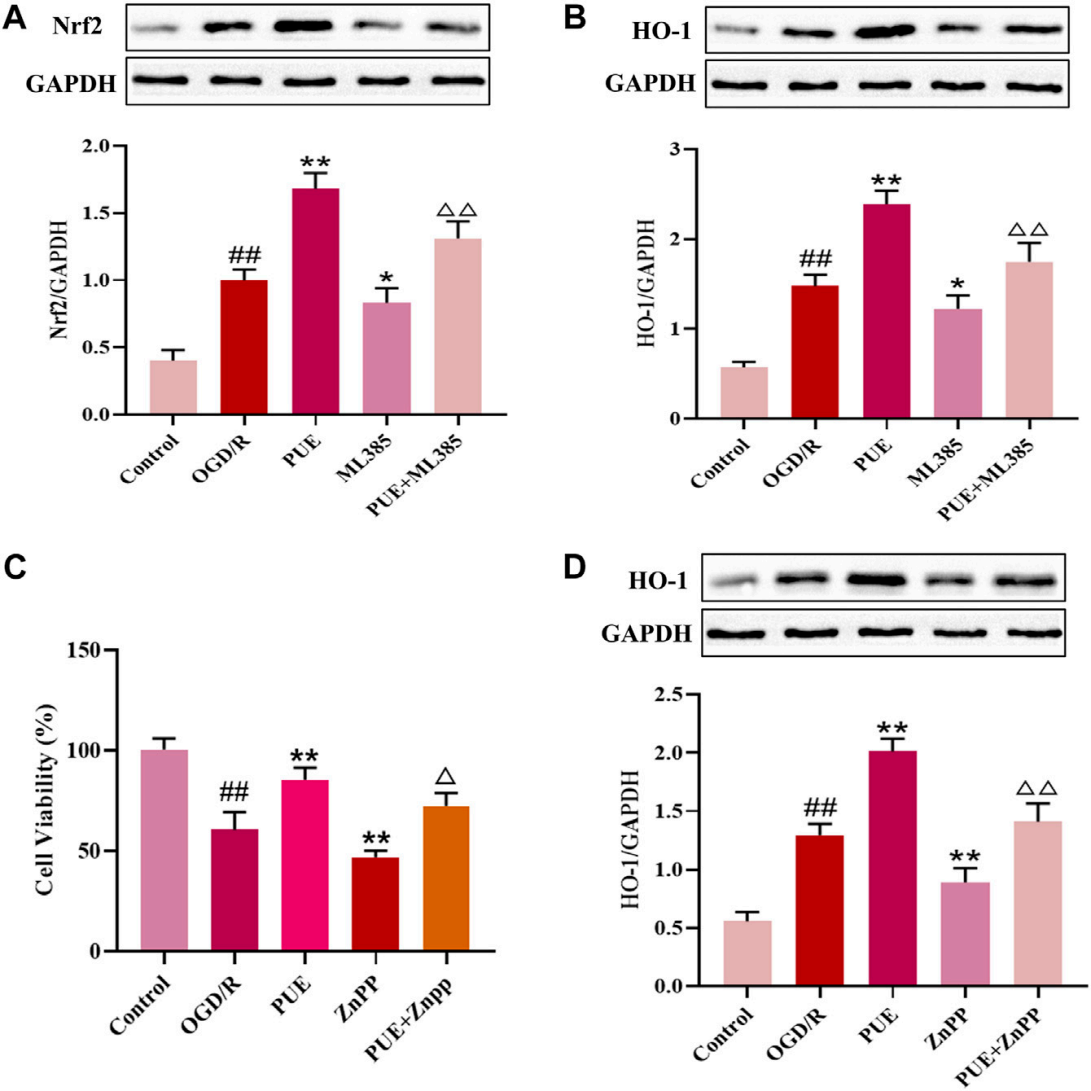
PUE promotes the upregulation of HO-1 expression by activating Nrf2, and HO-1 inhibitor ZnPP reverses the protective effect of PUE. **(A)** Protein expression of Nrf2 after administration of ML385. **(B)** Protein expression of HO-1 after administration of ML385. **(C)** Cell viability after ZnPP administration. **(D)** Protein expression of HO-1 after administration of ZnPP. All data are expressed as mean ± SD. ^##^ *p* < 0.01 vs. control group, *p < 0.05 or **p < 0.01 vs. OGD/R group, ^△^ *p* < 0.05 or ^△△^ *p* < 0.01 vs. PUE group.

In this study, we observed the expression of Nrf2 (green marker) and the cell nucleus (blue marker) under laser confocal microscopy (Figure 8A). Green fluorescence was more intense in the OGD/R group than in the control group, and the fluorescence intensity was significantly increased after PUE treatment. The result showed that Nrf2 is activated by OGD/R and PUE treatment further promotes nuclear translocation. The Western blotting results showed no trend in PI3K and Akt expression, but p- PI3K, p-Akt, cytoplasm Nrf2, and Keap1 expression decreased (*p* < 0.01). On the contrary, nucleus Nrf2 and HO-1 increased (*p* < 0.01) after OGD/R. Meanwhile, compared with the OGD/R group, cytoplasm Nrf2 and Keap1 expression increased, p-PI3k, p-Akt, nucleus Nrf2, and HO-1 expression decreased in the LY294002 group (Figures 8C–I). However, the above phenomenon could be reversed by PUE. The co-immunoprecipitation results showed that PUE could promote the dissociation of Nrf2 from Keapl, and further verified the mechanism of Nrf2 activation by PUE under the condition of oxidative stress (Figure 8B). The relevant mechanism is shown in Figure 9.

### 3.9 PUE promotes the upregulation of HO-1 expression by activating Nrf2, and HO-1 inhibitor ZnPP reverses the protective effect of PUE

**FIGURE 10:**
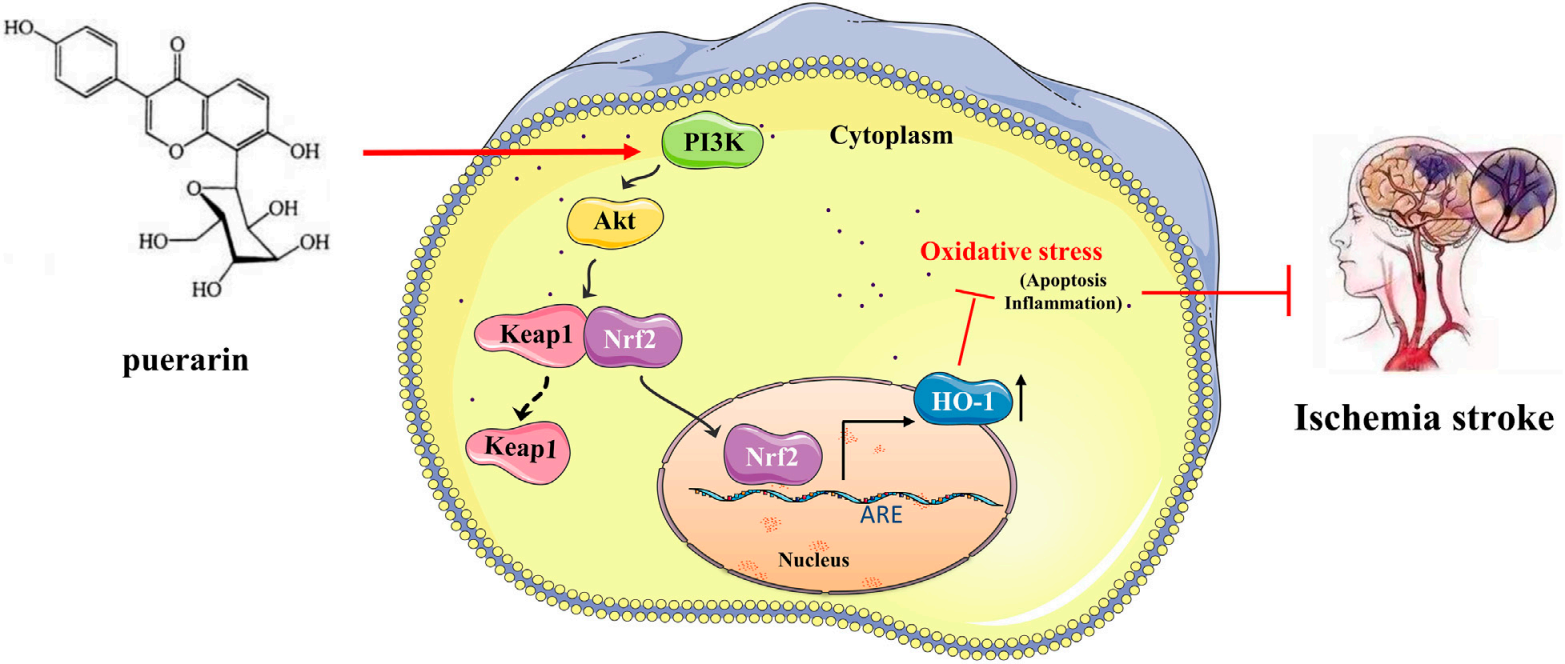
Puerarin can reduce oxidative stress by activating PI3K/Akt/Nrf2 pathway to achieve neuroprotective effect on ischemic stroke.

To further investigate whether PUE upregulates HO-1 expression by activating Nrf2, we administered the Nrf2 inhibitor ML385. As shown in Figures 9A, B, PUE upregulated Nrf2 expression, ML385 significantly decreased Nrf2 expression, and there was a decreasing trend of HO-1 expression level with the decrease of NRF2 expression. The results suggest that PUE may increase the expression of HO-1 by activating Nrf2. We next intervened HT22 cells with HO-1 inhibitor ZnPP to observe the changes in cell viability. The results in Figure 9C show that PUE administration largely restores the damage caused by OGD/R, while the ZnPP inhibitor causes a significant decrease in cell viability. In Figure 9D, it can be seen that PUE can upregulate HO-1 expression after OGD/R to further exert antioxidant effects, while HO-1 expression significantly decreased after ZnPP administration, indicating that ZnPP can reverse the protective effect of PUE on OGD/R-injured HT22 cells. Figure 10.

## 4 Discussion

I/R usually occurs after the sudden interruption of blood flow to the cerebral ischemic area which is an effective means of reducing disability (Herpich and Rincon, 2020). But at the same time, I/R will also bring adverse factors such as neuronal apoptosis and oxidative stress to the brain. Oxidative stress is one of the pathological mechanisms of I/R, which exacerbates the injury to neuro cells based on numerous types of research (Deng et al., 2022; Hu et al., 2022). Thus, alleviating oxidative stress and protecting injured nerve cells is the main strategy to treat IS.

PUE is a monomeric component with anti-I/R effects from traditional Chinese herbs (Lian et al., 2019). However, whether PUE exerts therapeutic effects on I/R by inhibiting oxidative stress has not been clearly investigated. The present study is the first to elucidate the mechanism of the protective effect of PUE on I/R through *in vivo* and *in vitro* models. It was confirmed that PUE reduced brain histopathological alterations in MCAO/R rats. PUE could also protect the HT22 cells from OGD/R. The mechanism of regulating the oxidative stress effect of PUE was further investigated by HT22 cells injured by OGD/R related to PI3K/Akt/Nrf2 signal pathway.

I/R leads to massive production and difficult scavenging of free radicals such as ROS, with consequent oxidative stress (Du et al., 2019; Gao et al., 2020; Shao et al., 2020). The overproduced ROS could induce the apoptosis of neuronal cells and ROS was selected as the core marker to evaluate the therapeutic effect of PUE. LDH is another essential indicator to assess the degree of I/R injury, which remarkably increased in OGD/R injured neuro cells. According to this study, the level of ROS was downregulated by PUE. Confirmation that the protective effect of PUE on I/R is largely derived from scavenging free radicals.

Next, the anti-apoptotic effect of PUE on neuronal cells was studied. The results presented that the apoptosis rate of MCAO/R model rats and the injured HT22 cells were both reduced after being treated with PUE. Additionally, apoptosis is associated with Bcl-2, cleaved-caspase 3, and Bax. PUE treatment significantly decreased Bax/GAPDH ratio and increased the Bcl-2/GAPDH ratio according to our study, which indicated that the neuro cell apoptosis was reduced by PUE, thereby exerting its therapeutic effect.

More and more evidence showed that SOD, GSH, and GSH-px were the representative makers associated with oxidative stress (Zhou et al., 2019; Yu et al., 2022). Obviously, the level of SOD, GSH, and GSH-px were reduced in both MCAO/R and OGD/R models, whereas PUE treatment, significantly improved SOD, GSH, and GSH-px expression. Our results suggested that the protective effect of PUE on I/R by inhibiting oxidative stress. The proliferation and survival of cells are regulated by the PI3K/Akt. The phosphorylation of proteins associated with PI3K/Akt pathway is known to inhibit cell apoptosis and promote cell growth (Qi et al., 2014). Western blot results showed that MCAO/R and OGD/R significantly decreased the phosphorylation of PI3K and Akt, and this phenomenon can be reversed by PUE. Nrf2 is not only the downstream gene of PI3K/Akt but also an important transcriptional activator in the cytoplasm. Previous reports showed that activating PI3K/Akt pathway can stimulate Nrf2 transfer to the nucleus to release antioxidant enzymes and further reduce the ROS level (Bereczki et al., 2018; Liu et al., 2019). In addition, Keap1 and Nrf2 form a stable polymer in the resting state. Under external stimulation, Nrf2 is separated from the polymer, dissociates into the nucleus and binds to the antioxidant response element (AREs) to activate downstream HO-1, SOD and GSH (Liu et al., 2019). Consistent with previous research results, the expression of nucleus Nrf2 and HO-1 were also elevated when after being treated with PUE (Miao et al., 2022). Our results revealed that the therapeutic mechanism of PUE on IS was related to PI3K/AKT/Nrf2 pathway.

LY294002, was used to further confirm whether the action mechanism is related to PI3K/Akt/Nrf2 signal pathway. Interestingly, the expression of p-PI3K, p-Akt, nucleus Nrf2, and HO-1 were inhibited by LY294002 in both *in vivo* and *in vitro* experiments. Furthermore, protein interactions between Nrf2 and Keap1 were observed by co-immunoprecipitation experiments, and we found that PUE induced dissociation of the Nrf2-Keap1 complex and accelerated nuclear translocation of Nrf2, suggesting that PUE does reduce oxidative stress by activating the PI3K/Akt/Nrf2 signaling pathway. Finally, by administering Nrf2 inhibitor and HO-1 inhibitor, we found that the decrease in Nrf2 expression was accompanied by a decrease in HO-1 expression, demonstrating that PUE may exert antioxidant effects by activating Nrf2 to promote upregulation of HO-1 expression, while HO-1 inhibitor was able to reverse the protective effect of PUE on OGD/R-injured HT22 cells.

Besides, we also evaluated the cytotoxicity of PUE on HT22 cells. The results showed that PUE had no damage to HT22 cells when the concentration was lower than 250 nmol/L. The results show that PUE was safe within a certain concentration.

## 5 Conclusion

Overall, it is the first time to investigate the mechanism of protective effect of PUE via antioxidant function on I/R-induced neuronal injury. Our experimental data suggest PUE had a significant neuroprotective effect both *in vivo* and *in vitro*, which is possible association with the PI3K/Akt/Nrf2 signaling pathway. Our present study also provided a new way to treat cerebral ischemia.

## Data Availability

The original contributions presented in the study are included in the article/Supplementary Material, further inquiries can be directed to the corresponding authors.

## Abbreviations

Akt, protein kinase B; CCA, common carotid artery; CCK8, Cell Counting Kit-8; HE, Hematoxylin and Eosin; HO-1, Heme oxygenase-1; I/R, Ischemia/reperfusion; ICA, internal carotid artery; MCAO/R, Middle cerebral artery occlusion/reperfusion; Nrf2, Nucleus factor erythroid 2-related factor 2; OGD/R, Oxygen-glucose deprivation/reperfusion; PI3K, Phosphoinositide 3-kinase; PUE, Puerarin; ROS, Reactive oxygen species; TTC, 2, 3, 5-triphenyl tetrazolium chloride.
